# Reproductive and Flight Characteristics of *Lymantria xylina* (Lepidoptera: Erebidae) in Fuzhou, China

**DOI:** 10.3390/insects15110894

**Published:** 2024-11-15

**Authors:** Jifeng Zhang, Baode Wang, Liqiang Wang, Cheng Zuo, Junnan Li, Yonghong Cui, Xuanye Wen, David Cowan, Songqing Wu, Mengxia Liu, Rong Wang, Feiping Zhang

**Affiliations:** 1 College of Forestry, Fujian Agriculture and Forestry University, Fuzhou 350002, China; zhangjif118@163.com (J.Z.); fpzhang1@163.com (F.Z.); 2 US Department of Agriculture, Animal and Plant Health Inspection Service, Science and Technology, Forest Pest Methods Laboratory, Buzzards Bay, MA 02542, USA; 3 Fujian Academy of Forestry Sciences, Fuzhou 350012, China

**Keywords:** *Lymantria xylina*, female moth, reproduction, flight, circadian rhythm, dispersal risk

## Abstract

The biological characteristics of *Lymantria xylina* Swinhoe (Lepidoptera: Erebidae), a moth that threatens coastal forests in Fuzhou, China, are closely linked to its spread risk. To characterize these traits, we primarily investigated emergence, reproductive, and flight behaviors. Our findings show that females typically emerge, mate, and copulate during specific times of day. The peak hours of emergence, courtship, and copulation are 13:00–14:00, 19:00–21:00, and around 0:00, respectively. The starting time of oviposition was concentrated before dawn and during the daytime. They preferentially lay eggs on columnar objects, including artificial ones. On average, females laid 361 eggs, lived for 4.5 days, and weighed 0.74 g. Non-ovipositing females were observed to fly for short distances, especially during the evening. Field observations suggest that these females can potentially travel up to 184.5 m in total and 34.5 m continuously. While this indicates a theoretical risk of long-distance dispersal, our findings suggest that the overall risk is limited. These results contribute to our understanding of the biology and dispersal potential of *L. xylina*.

## Simple Summary

*Lymantria xylina* Swinhoe (Lepidoptera: Erebidae), a moth regulated as a potential invasive species by countries of the North American Plant Protection Organization (NAPPO), threatens coastal forests in Fuzhou, China. This insect spends most of its adult life focused on reproduction and flight, which are crucial for its survival and spread. We studied how these moths emerge from their cocoons, mate, lay eggs, and fly towards light. By understanding these behaviors, we can better predict how they might spread to new areas, and develop strategies to control their populations. This research provides valuable insights into the biology of this potentially invasive insect.

## 1. Introduction

*Lymantria xylina* Swinhoe is a significant pest of protected *Casuarina* spp. forests in southeastern China. It occurs in a large area in a *Casuarina* plantation along the coast of Fujian province of China and often causes serious infestation. The insect feeds on *Casuarina* twigs (i.e., leaves), which hinders the normal growth of *Casuarina* trees and accelerates their weakening, which may cause the whole tree to die [1,2]. The insect has many host species, and in recent years, it has also been reported to cause damage to trees such as *Betula alnoides* Buch.-Ham. *ex* D. Don, *Litchi chinensis* Sonn, and *Morella rubra* Lour [1,3]. *L. xylina* is primarily distributed in East Asia, including Japan, India, and regions of China such as Fujian, Taiwan, Guangxi, Guangdong, and Zhejiang [4,5,6]. *L. xylina* adults exhibit strong phototactic behavior, and some females can sometimes fly short distances, suggesting the possibility of being attracted to ships’ lights and subsequently landing on ships or cargo to lay eggs, resulting in long-distance movement [1,7,8]. Reports indicate that *L. xylina* poses a serious invasion risk to subtropical regions of the United States, such as Hawaii, Florida, and southern California [9,10]. Since 2015, small numbers of *L. xylina* adults or egg masses have been found on freighters or containers in several coastal ports of Korea [1,11,12,13].

In Fujian Province, China, *L. xylina* occurs one generation a year. The egg stage is generally up to 9 months; hatching begins in mid and late March of the following year and stops from mid to late April. The larval stage is usually 45–64 d, generally seven instars. The average pupal period is 10.7 days, starting mid-May and ending mid-June. Adults begin to emerge at the end of May, peak in early June, and end in late June. Adults begin to lay eggs at the end of May, and female moths usually lay only one egg mass, which is oyster-shaped and attached to the twigs. The damage period of *L. xylina* is short (from March to June), and biological, physical, artificial, and other comprehensive control measures can be coordinated according to its biological characteristics. The specific measures are as follows: (1) afforestation measures, planting Casuarina trees strains resistant to *L. xylina*, such as P_10–33_; (2) manual control, picking egg masses or killing newly hatched larvae; (3) physical control, using insecticidal lamps to trap and kill male moths in the peak period of emergence; (4) biological control, using *Beauveria bassiana* and *nucleopolyhedrovirus* to control larvae; (5) chemical control, applying low-toxicity, high-efficiency, low-residue chemical agents such as 25% chlorbenzuron at a 1000-fold dilution during outbreak periods to control larvae [14].

For a long time, as a species related to *Lymantria dispar* Linnaeus, *L. xylina* has been considered, similarly to *L. dispar*, a possible invasive pest because of its wide host range and similar biological characteristics. Therefore, the risk of its spread from its country of origin to other countries has attracted worldwide attention [1,8,15]. In mainland China, *L. xylina* is mainly distributed in the southeastern coastal area, and in theory, it has the species traits to spread to other regions through ocean-going freighters in southeastern coastal ports [2,10,16]. However, *L. xylina* is univoltine in the southeast coastal area of China. Its adult and pupal stages are short, and its larva is generally weak, and the mortality rate is high at various temperatures. For instance, they cannot survive at 33 °C and cannot pupate at 15 °C or 30 °C [17,18,19]. Consequently, the possibility of *L. xylina* dispersing via adults, larvae, or pupae on ocean-going vessel dispersal is low. Instead, *L. xylina* diapause eggs can survive both summer and winter, the egg stage can last up to 10 months in a year, and egg masses are highly durable [1,17]. Therefore, egg masses are considered the most suitable form for oceanic dispersal. However, egg masses are laid by females, which are attracted by light and fly to ships docked at ports or containers stored at ports. Thus, the reproductive and flight characteristics of adult *L. xylina* are crucial in evaluating the potential of transoceanic spread.

Despite the close relationship between the biological characteristics of adult *L. xylina* and its spread risk, comprehensive studies on these characteristics remain limited. In mainland China, only Zuo [20] and Li [21] have conducted relatively comprehensive studies on the biological characteristics of *L. xylina* adults. However, Zuo’s research focused mainly on emergence and reproductive behaviors, and its research goal was the olfactory differences between male and female antennae [20]. Li’s research was more comprehensive but provided only brief descriptions [21]. Regarding phototaxis and flight behavior, while *L. xylina* adults exhibit phototactic behavior in mainland China, there are no published reports on the phototactic behavior of females. Additionally, although females can fly short distances, there is a lack of data on flight phenomena or field tests of flight capabilities. To clarify the biological characteristics of *L. xylina* adults and discuss their spread risk, this paper summarizes the biological characteristics of adults related to spread risks based on the research on the biological characteristics of adults over the years 2016 to 2022. Using both observational and experimental methods, we conducted comprehensive studies of *L. xylina* adult emergence and reproductive behavior, as well as preliminary examinations of their flight behavior. The specific objectives of the study were (1) to evaluate whether there is a risk of *L. xylina* reaching freighters and spreading with the freighters by studying whether the female moths exhibit the ability of phototactic flight and lay eggs on objects; (2) to determine the law of spread through the behavioral circadian rhythm of *L. xylina* adults; and (3) to speculate the magnitude of spread risk based on reproductive and flight characteristics.

## 2. Materials and Methods

### 2.1. Emergence Experiments

#### 2.1.1. Observations of Emergence in the Laboratory

*Lymantria xylina* pupae were collected from a *Casuarina* plantation in Fuzhou City, Fujian Province, China (119°43′15.67″ E, 25°31′43.74″ N), on 2 June 2020. A total of 360 pupae were randomly selected and divided into three groups of 120 each. These were placed in indoor net cages (75 cm × 75 cm × 75 cm, 100 mesh nylon nets). To simulate airflow in natural conditions, which can stimulate *L. xylina* adults mating, the room was ventilated with open doors and windows, but without artificial lighting, or temperature or humidity control. The temperature ranged from 25.4 °C to 30.3 °C, and the relative humidity (RH) ranged from 64% to 89%. Newly emerged adults were weighed individually and placed in transparent plastic boxes (30 cm × 24 cm × 10 cm). The number, sex, and emergence times were recorded hourly until all adults had emerged. After emergence, adults were categorized as follows: 1-day-old was 0–24 h after emergence, 2-day-old was 24–48 h after emergence, and so on. In addition, the difference in emergence time between males and females was calculated by the weighted average method:

M_difference_ (day) = M_♀_ (female emergence) − M_♂_ (male emergence)

M (day) = (x_1_ × 1 + x_2_ × 2 + … + x_n_ × n)/(x_1_ + x_2_ + … + x_n_)

where x_n_ is the number of adults that emerged on the nth day from the first day of emergence.

#### 2.1.2. Dynamics of Emergence in the Forest

To monitor the emergence period and number of adult male *L. xylina*, five milk carton traps (Figure S1) were deployed in a *Casuarina* plantation in Fuzhou (119°48′21.76″ E, 25°36′56.45″ N) from 23 May to 27 June 2017. The lure we used was xylinalure (*cis*-7, 8-epoxy-2-methyleicosane), which was rope-shaped, 12 cm long, and 2 mm in diameter. It was valid for 180 days in the field. A Vaportape II strip (DDVP, Hercon Environmental, Emigsville, PA, USA) was also placed inside the trap. Traps were hung at 2 m high, spaced approximately 50 m apart. Trapped male moths were collected approximately every seven days, and their numbers were counted.

### 2.2. Reproductive Behavior Experiments

#### 2.2.1. Observation of Reproduction in the Laboratory

The source of the moth was the same as that in Section 2.1.1. Healthy female and male moths that emerged on the same day were randomly paired and placed in transparent plastic boxes with a *Casuarina* twig inside to observe their courtship, mating, and oviposition behavior. A total of 49 pairs were observed with hourly checks. We recorded the number, start time and end time of courtship, mating and oviposition behaviors of female moths, as well as the death time of adults and the number of egg masses. Egg masses were decomposed to count the number of eggs. Female moths were considered to exhibit courtship behavior when their tails were continuously twisted and their ovipositors were continuously retracted [20,22,23].

#### 2.2.2. Observation of Copulation in the Forest

To observe the mating behavior of adult *L. xylina*, a *Casuarina* plantation in Fuzhou (119°42′48.05″ E, 25°31′51.71″ N) was monitored for four consecutive days from 7 to 10 June 2021, and the main observation time period was 23:00–1:00. A total of 28 pairs of adults were observed to be mating.

### 2.3. Oviposition Behavior Experiments

#### 2.3.1. Observation of Oviposition Dynamics Outdoor

To study oviposition dynamics, wild-caught females that had not yet laid eggs were placed on small *Casuarina* twigs fixed to foam boards starting at 11:00 on 3 June 2022. Each twig held one female, and there were 13 branches. We collected the eggs laid by each female moth every hour (peeling off the egg masses from the twigs and collecting the scattered eggs) for three days. The duration of one oviposition event was determined by calculating the number of eggs laid hourly. A continuous oviposition period was defined as a period where eggs were laid every hour without interruption. If eggs were laid every hour for a continuous period, it was considered as one oviposition event.

#### 2.3.2. Observation of Oviposition Conditions

To study the impact of oviposition conditions, newly emerged females and males of *L. xylina* with similar body sizes were selected and placed in pairs in transparent plastic boxes, with two groups, each containing nine pairs. One group was provided with *Casuarina* twigs for oviposition, while the other was not provided with *Casuarina* twigs. The environment was maintained at 28 °C, 80% RH, and in complete darkness. Observations were made hourly; the number, duration of oviposition behavior, and the number of egg masses were recorded. At the end of the experiment, egg masses were dissected, the number of eggs was counted, and the fecundity of each female moth was classified as high (more than 200 eggs), low (fewer than 200 eggs), or none (no eggs).

#### 2.3.3. Observation of Oviposition Object Preference

To investigate oviposition object preference, steel plates (60 cm × 10 cm) were smeared with six paints (red, yellow, black, white, green, and blue), and after the steel plates were dried, they were placed around the net cage (75 cm × 75 cm × 75 cm, 100 mesh nylon nets) (Figure S2). A total of 30 pairs of *L. xylina* adults were released into the cage on 7 June 2016, and oviposition was observed under the same environmental conditions as described in Section 2.1.1.

In 2017, the attachment of *L. xylina* egg masses to non-plant objects, such as soil, rocks, walls, doors, windows, and utility poles, was investigated at four sites in Fuzhou (Figure S3). Five sample plots (each sample area: 10 m × 10 m) were taken from each site. Additionally, the substrates of egg masses on artificial objects in the laboratory (Key Laboratory of Integrated Pest Management in Ecological Forests of Fujian Provincial Universities, Fujian Agriculture and Forestry University, Fuzhou, Fujian, China) were also recorded. In the laboratory, adult experiments were mainly carried out in net cages (for multiple adults) and plastic boxes (for a pair of adults or one adult). Iron cages were occasionally used to observe adults, and bricks and foam were occasionally used in cages to fix larger branches.

### 2.4. Flight Experiments (Including Phototactic Flight)

#### 2.4.1. Observation in Cage Outdoor

To study flight behavior, ten non-ovipositing *L. xylina* females were placed in a net cage (75 cm × 75 cm × 75 cm, 100 mesh nylon) located on the northern side of an eave near a *Casuarina* plantation at 14:00 on 3 June 2022. The entire process was recorded by a video camera (SONY HDR-SR12E, Sony Group Corporation, Minato City, Japan) and illuminated by red lights at night. After the female moths ceased flying, the recording was ended, and the first wingbeat preheating duration, the number of flights per hour, and the flight duration per hour of each female moth were counted.

#### 2.4.2. Tracking and Observation in the Forest

As male *L. xylina* do not lay eggs and are difficult to track due to their high flight speed, only eight females were observed and tracked. On 5 June 2022 (sunny, 25~29 °C), tracking and observation began at 14:00. With an action camera (DJI Osmo Action 1, Shenzhen DJI Technology Co., Ltd., Shenzhen, China) worn on the head, we searched for female *L. xylina* in the *Casuarina* plantation in Fuzhou (119°42′48.05″ E, 25°31′51.71″ N). The camera was used to record flight duration and behavior. When a non-ovipositing female moth was found, the branch or trunk on which the female moth was resting was continuously beaten with a branch (Figure S4). The female was followed and filmed after being frightened and taking flight. Flight distance and total flight distance of the female were recorded by the Route Assistant app (a mobile app for recording outdoor mileage and tracks, Shenzhen 2bulu Information Technology Co., Ltd., Shenzhen, China), and flight height was estimated. After landing, the female moth was scared and tracked again until it ceased flying. Other flight-related phenomena such as the flight process (takeoff, flight status, and landing) of the female moth, the movement behavior of the female moth during and after oviposition, were also observed and recorded.

#### 2.4.3. Verification of Phototactic Flight Behavior in the Laboratory

On 5 June 2020, a test was conducted in a room (6.6 m × 6.0 m × 3.0 m) with an electric insecticide lamp (main peak wavelength: 365 nm; power: 15 watts; base height: 35 cm; lamp model: GP-LH18B; Zhejiang LOIHOI Agriculture and Forestry Technology Co., Ltd., Taizhou, China) as the light source. The lamp was placed in the center of the room, and the cage (75 cm × 75 cm × 75 cm) containing indoor-emerged adults was placed 3.0 m away from the light source. The lamp was turned on from 23:00 to 6:00, and the movement of male and female moths of *L. xylina* towards the lamp was continuously observed. Two tests were conducted: Test 1 involved ten virgin females (1–2 days old, unmated), and Test 2 involved mixed-age males and females (1–3 days old, mating status unknown, female non-ovipositing), with ten individuals of each sex tested.

#### 2.4.4. Marking–Release–Recapture Tests in the Field

This experiment was conducted in Fuzhou (119°43′57.98″ E, 25°33′11.88″ N) in an open sandy area measuring approximately 90 m north to south and 3000 m east to west, with no trees on the eastern and western sides and two rows of *Casuarina* trees on the northern and southern sides. The insecticidal lamp was the same as that described in Section 2.3.3 and was hung at a height of 2 m. The test insects were male and female of *L. xylina* (1–3 days old, virgin, non-ovipositing). From 19:00 to 4:00 on 15–17 June 2021 (cloudy, 27–31 °C), 90 tagged male moths and 45 female moths, tagged by waterproof marker pens with different colors, were released only once. All individuals were released on three days from different distances (30, 60, and 90 m) from the insecticide lamp (located on the northern side) and three different orientations (eastern, western, and southern) within each trial. For example, on 15 June, ten male and five female moths were released 90 m from the insecticide lamp in the eastern direction of the test site. The recapture situation was checked for three consecutive days.

### 2.5. Data Analysis

Data were analyzed using SPSS 22.0 software (IBM, Armonk, NM, USA). Data are expressed as the mean ± standard error. After the data met the assumptions of normality and homogeneity of variance, the comparative analysis was carried out. For comparisons between two groups, independent samples *t*-testing was used. For comparisons involving more than the two groups, analysis of variance (ANOVA) was followed by Duncan’s multiple range test to identify significant differences between specific groups.

## 3. Results

### 3.1. Emergence Experiments

#### 3.1.1. Observations of Emergence in the Laboratory

A total of 184 adults emerged, with an emergence rate of 51.1%. This included 61 females and 123 males, resulting in a sex ratio of approximately 0.33. Prior to emergence, the top of the head of the pupae cracked open slightly, and the body twisted continuously as the crack expanded. The adult moth then emerged, followed by wing hardening, slowly stretching, and fluttering, accompanied by the discharge of brown waste fluid from the tail. This process took from several minutes to an hour.

**Figure 1:**
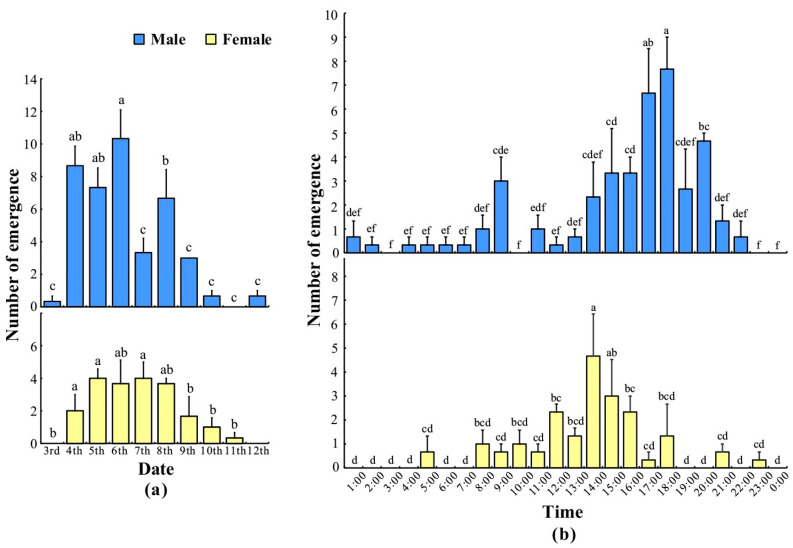
Changes in the number of emerged male and female *L. xylina* adults in the laboratory in 2020: (**a**) the daily change in the number of emerged male and female moths; (**b**) the change in the number of emerged male and female moths per hour. The data in the figure are the mean ± SE; different lowercase letters represent significant differences in the number of emerged male or female *L. xylina* adults (*p* < 0.05, Duncan’s test).

As shown in Table 1, time and date had extremely significant effects on the emergence of female and male *L. xylina*, respectively, but time × date had no significant effect on the emergence of female moths (*p* = 0.196). Figure 1a illustrates the daily emergence variation in *L. xylina* adults. Males emerged first, with a rapid increase in numbers, reaching a peak on 6 June. Subsequently, there was a sharp decline, significantly lower than the emergence numbers from 4 to 6 June. Females emerged later, with a more stable number variation, peaking on 7 June, and then gradually decreasing. According to the weighted average method, male moths emerged approximately 0.5 days earlier than female moths.

The circadian rhythm of the emergence of *L. xylina* adults is shown in Figure 1b. Generally, both male and female moths emerged mainly between 4:00 and 21:00, accounting for 91.2% of the total emergence. Male moths could emerge at almost any time, with the highest proportion emerging between 17:00 and 18:00 (18.7%), followed by 16:00 to 17:00. Emergence was minimal between 22:00 and 3:00, indicating that male moths primarily emerged in the afternoon and evening, with a peak in the evening. Female moths had the highest emergence between 13:00 and 14:00 (23.0%), followed by 14:00 to 15:00, with minimal emergence between 18:00 and 7:00. This suggests that female moths primarily emerged during the day, with a peak in the afternoon.

#### 3.1.2. Dynamics of Emergence in the Forest

**Figure 2:**
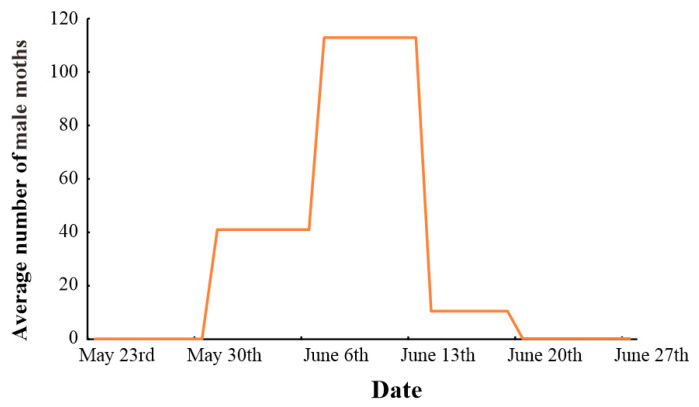
Emergence dynamics of male *L. xylina* moths in the forest in Fuzhou, China, in 2017.

The dynamics of the emergence of male *L. xylina* in a *Casuarina* plantation in Fuzhou in 2017 are shown in Figure 2. The emergence period lasted about 35 days, with the initial period from 23 May to 30 May (total of one male trapped) and the final period from 19 June to 27 June (total of two males trapped). The emergence period of *L. xylina* adults was concentrated and overlapping, with an initial peak period, the peak period, and the late peak period. The peak period occurred approximately from 8 June to 12 June, after which the number of male moths in the forest rapidly decreased, entering the late peak period.

### 3.2. Reproductive Behavior Experiments

#### 3.2.1. Observation of Reproduction in the Laboratory

##### Courtship Behavior

**Figure 3:**
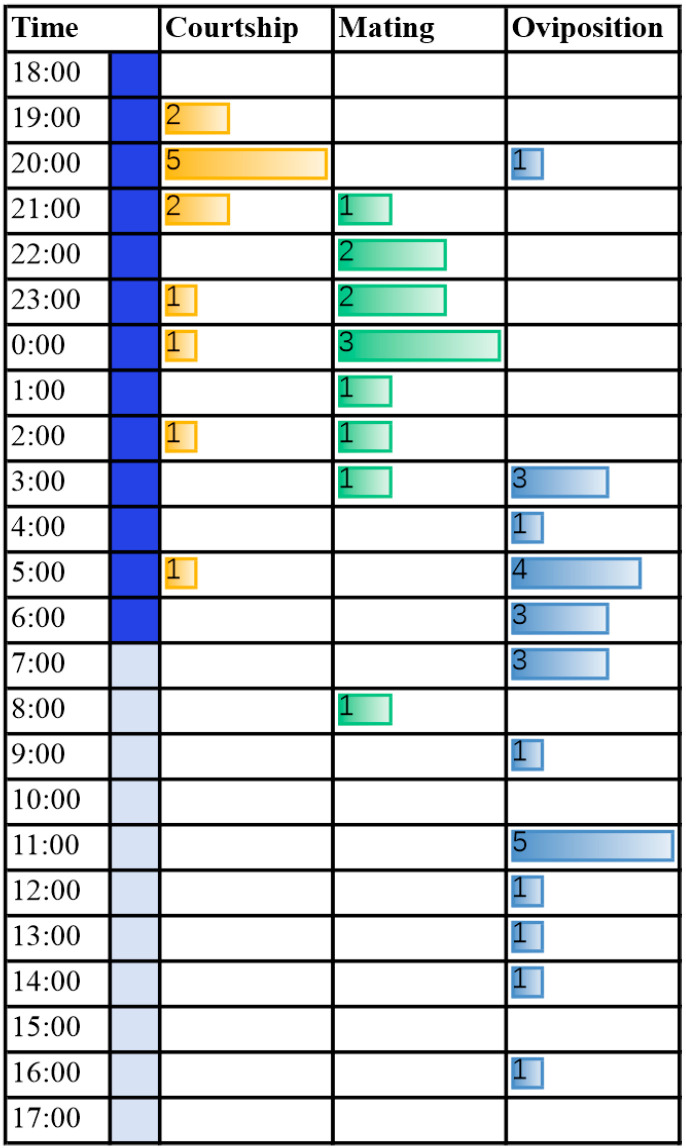
Circadian rhythms of courtship, mating, and ovipositing behavior of *L. xylina* adults in the laboratory in 2020.

Female *L. xylina* moths began courtship behavior on the day of emergence, with the pre-courtship period (from emergence to the start of courtship behavior) ranging from 3.9 h to 15.0 h, averaging 8.4 h. During courtship, the female moths constantly twisted their tails and retracted and extended their ovipositors, releasing sex pheromones to attract male moths. Upon detecting the sex pheromones, male moths became excited, fluttered their wings, and crawled or flew around the female moths, extending, bending, and exploring with their genitalia in an attempt to copulate. The courtship period (from the start to the end of courtship behavior) ranged from less than 1.0 h to 22.2 h, averaging 5.4 h. Among 49 observed pairs of adults, 13 pairs of female moths displayed courtship behavior, which occurred after 19:00 and peaked around 20:00, accounting for 69.2% of the total (Figure 3).

##### Copulation Behavior

Copulation behavior was observed in 12 pairs of *L. xylina* moths, primarily occurring at night, concentrated around midnight, with occasional daytime copulation (Figure 3), which indicates that midnight is the peak period for *L. xylina* copulation. Female moths can copulate on the day of emergence, with the pre-copulation period (from emergence to the start of copulation) ranging from 7.4 h to 38.0 h and averaging 16.9 h. During copulation, both male and female moths remained motionless, and the duration of copulation ranged from less than 1.0 h to 5.1 h, averaging 3.6 h. There are three main copulation postures of *L. xylina*: end-to-end, venter-to-venter (i.e., the abdomens of the male and female moth face each other), and male on top with female dorsum up (Figure S5). Among these, the male on top with female dorsum up position was the most common indoor copulation posture (Figure S5c).

##### Oviposition Behavior

As shown in Figure 3, oviposition behavior was observed in 25 female moths, primarily occurring in the early morning and daytime (96.0%), demonstrating significant diurnal rhythm. The female moth remained motionless after copulation for a minimum of 1.0 h, a maximum of 11.9 h, and an average of 5.1 h, before initiating oviposition. Most eggs were laid on *Casuarina* twigs, and a few were laid on transparent plastic boxes.

During oviposition, female moths’ wings formed a cross shape with the abdomen contracted under the wings, and the tail of the abdomen bent downwards and swung left and right, laying eggs while secreting white adhesive liquid. The outer layer of the egg mass was covered with brown fuzz. Freshly laid eggs were milky white, turning brown shortly after. The oviposition duration ranged from 5.0 h to 139.5 h, averaging 46.8 h. Some female moths, placed singly in a plastic box, laid eggs without mating. Some female moths died before completing oviposition or survived after finishing oviposition. The egg masses laid on *Casuarina* twigs were mostly long and oyster-shaped, while those laid on transparent plastic boxes were nearly round. The egg masses were hard, with tightly packed eggs and a few covered with fuzz, and were closely attached to the oviposition location.

##### Adult Weight, Lifespan, and Egg Production

The weight ranges of female and male moths were 0.27–1.30 g and 0.07–0.30 g, respectively, with average weights of 0.74 g and 0.16 g. This indicates greater variability mainly in female weights (Table 2). The lifespan of female moths ranged from 1 to 10 days, averaging 4.5 days, while male moths lived for 1 to 9 days, averaging 4.9 days. Among the 49 observed female *L. xylina* moths, 40 females laid eggs, with an egg-laying rate of 81.6%. Females laid 1–4 egg masses indoors, with some laying fragmented or scattered eggs. Of these, twenty-eight females laid only one egg mass each (70.0%), and eight females laid two egg masses each (20.0%). The egg masses were dissected, revealing an egg count per female ranging from 12 to 1330 eggs, with an average of 361 eggs.

#### 3.2.2. Observation of Copulation in the Forest

**Figure 4:**
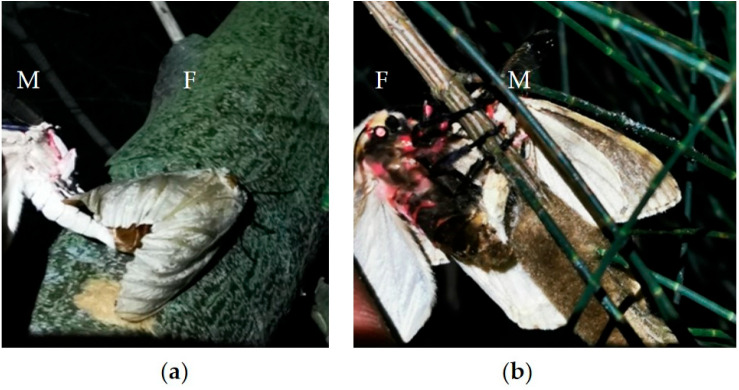
Females copulated secondarily during the oviposition interval: (**a**) initial oviposition; (**b**) late oviposition; F and M represent female and male moths, respectively.

The venter-to-venter pattern was the most common copulation posture in the forest (Figure S5b). Additionally, the phenomenon of two or three male moths aggregating around one female moth was observed in the forest (Figure S6). Secondary copulation, where female moths mated with male moths during oviposition intervals, was also observed (Figure 4a,b). From 7 June to 10 June 2021, a total of 28 pairs of adults were observed to be mating over four consecutive nights, with nine pairs (32.1%) involving secondary copulation.

### 3.3. Oviposition Behavior Experiments

#### 3.3.1. Observation of Oviposition Dynamic Outdoors

**Figure 5:**
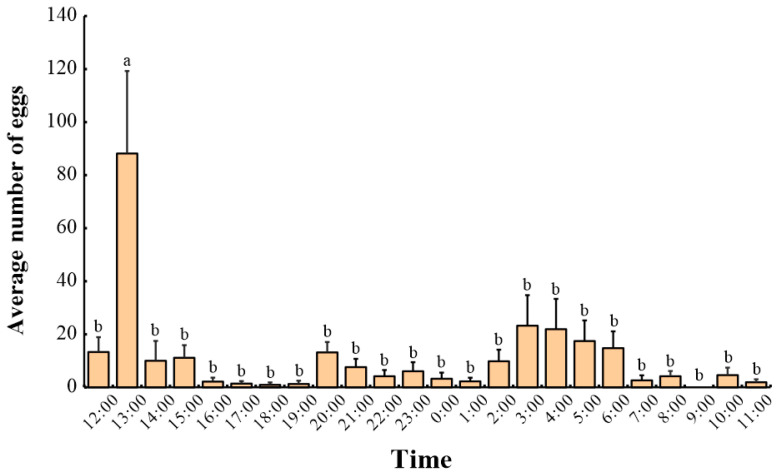
The time rhythm of female *L. xylina* adults laying eggs outdoors in Fuzhou in 2022: the data in the figure are the mean ± SE; different lowercase letters represent significant differences in the number of eggs laid in each period (*p* < 0.05, Duncan’s test).

As shown in Figure 5, oviposition by female moths was primarily concentrated during the first continuous oviposition period (from 12:00 to 15:00), accounting for 46.2% of the total. Each female moth laid eggs 1 to 11 times, averaging 5.5 times. The number of eggs laid per time ranged from 1 to 337, averaging 63. The maximum number of eggs laid at one time ranged from 14 to 337, averaging 223, accounting for 69.1% of the total egg count. The hourly egg-laying rate ranged from 1 to 293 eggs, averaging 28 eggs. The longest single oviposition duration ranged from 1 to 11 h, averaging 4.7 h. *L. xylina* oviposition was relatively concentrated, with most eggs laid during the first continuous oviposition period. After this period, oviposition became intermittent, with decreasing oviposition time and egg numbers.

#### 3.3.2. Observation of Oviposition Conditions

As shown in Table 3, among the nine pairs of *L. xylina* provided with *Casuarina* twigs for oviposition, six females laid eggs, each laying one long oyster-shaped egg mass on the twigs. Of these, three females laid more than 200 eggs, while the other three laid fewer than 200 eggs. Among the nine pairs without *Casuarina* twigs, only three females laid eggs. One female laid two nearly round egg masses on the bottom of the transparent plastic box, with more than 200 eggs. The other two laid fewer than 200 eggs, with one female laying six irregularly shaped egg masses. The average numbers of eggs per group were 429 and 164, with oviposition durations of 21.0 and 4.3 h, respectively, showing significant differences. Overall, egg masses laid on the bottom of the transparent plastic boxes were fragmented, scattered, and fewer in number. Some females exhibited oviposition behavior without laying eggs.

#### 3.3.3. Oviposition Preference on Artificial Objects

**Figure 6:**
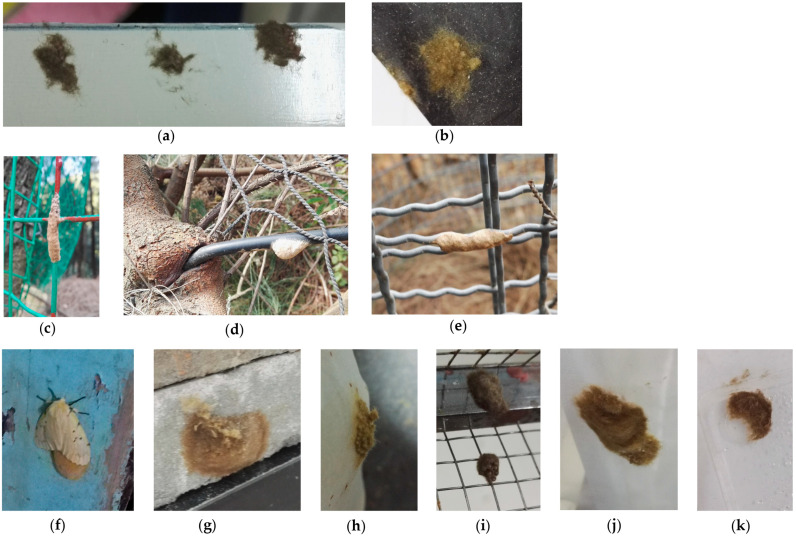
The oviposition phenomena of *L. xylina* on artificial objects: (**a**) white steel plate and (**b**) black steel plate were the results of a simulated cabin oviposition test in 2016; (**c**) green isolation net, (**d**) black wire, and (**e**) silver–gray wire were the oviposition phenomena of *L. xylina* on artificial objects in the natural environment from 2017 to 2022; (**f**) wooden door, (**g**) brick, (**h**) nylon mesh, (**i**) wire mesh, (**j**) foam, and (**k**) transparent plastic were the oviposition phenomena of *L. xylina* on artificial objects in the experimental environment from 2017 to 2022.

As shown in Figure 6a,b, in this simulated ship’s cabin oviposition experiment, six egg masses were laid on white steel plates (two on the front, and four on the back), and two egg masses were laid on black steel plates (both on the front). However, these egg masses were fragmented, small in size, and few in number, with some scattered eggs. No egg masses were laid on the other four types of steel plates. However, 16 egg masses were laid on the nylon mesh of the rearing cage.

In the field, no egg masses were found on non-plant surfaces within the experimental plots. However, from 2017 to 2022, in the non-experiment plots, egg masses were occasionally found on artificial objects, such as green safety nets (about 30 times), black wires (once), and silver–gray wires (once). These substrates were all cylindrical objects with diameters less than 1 cm (Figure 6c–e), and no egg masses were found on flat surfaces. These results indicate that *L. xylina* females could lay eggs on artificial objects. Among the artificial objects investigated, *L. xylina* showed a preference for cylindrical objects with diameters less than 1 cm.

In addition, as shown in Figure 6f–k, in other experimental environments, female moths laid eggs on wooden doors (once), bricks (once), foam (once), fine wires (multiple times), nylon mesh (numerous times), and transparent plastic (numerous times). Among them, the wooden door was in a light-trapping environment, while the others were in laboratory rearing environments (net cage).

### 3.4. Flight Experiments (Including Phototactic Flight)

#### 3.4.1. Observation in Cage Outdoor

The wingbeat preheating duration for the first flight of female moths ranged from 1.17 to 2.95 min, averaging 1.75 ± 0.18 min (*n* = 10). The duration per flight ranged from 0.03 to 13.00 min, averaging 1.45 ± 0.18 min (*n* = 113). This indicates that female moths require a long preheating period before taking off, with relatively short continuous flight durations.

**Figure 7:**
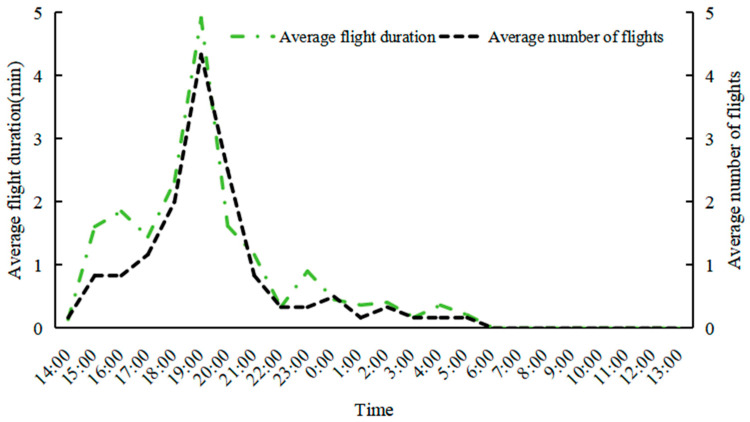
Diurnal rhythm of flight performance of female *L. xylina* moths in an outdoor cage in Fuzhou in 2022.

As shown in Figure 7, the flight duration and number of flights of female moths exhibited consistent variations, with clear rhythmic patterns. From 14:00 to 20:00, both the flight duration and number of flights rapidly increased, reaching a peak (average number of flights: 4.3 times; average flight duration: 4.9 min). After 21:00, both the flight duration and the number of flights rapidly decreased, and by 6:00, all female moths had begun oviposition and ceased flying, occasionally flapping their wings while crawling.

#### 3.4.2. Tracking and Observation in the Forest

Eight non-ovipositing female *L. xylina* moths were tracked in the field, with only four flying after the attached branches were tapped. As shown in Table 4, the total flight distance could reach 184.5 m, with a maximum uninterrupted flight distance of up to 34.5 m. The flight height was below 3.5 m and mostly between 0.2 and 1.0 m. Non-ovipositing females commonly flew at about 0.4 m above the ground before quickly descending or landing on *Casuarina* trees. Field tracking observations indicated that some non-ovipositing *L. xylina* females could perform short-distance flights, primarily in short bursts and with low flight heights.

The flight process of female moths typically involved warming up the wing muscles by fluttering their wings to raise their body temperature in preparation for takeoff, then gradually ascending with an increasing angle relative to the ground, followed by undulating flight resembling a wave pattern. Finally, they descended while flapping their wings, landing on the ground or attached objects. Female moths’ flight was characterized by long preparation times, quick descents, and short continuous flight distances, requiring rapid and continuous wing flapping during flight.

Between 2016 and 2022, we had the following observations: a smaller female moth was observed flying quickly over about 30 m at a height of about 5 m, landing in a tree canopy. Additionally, ovipositing females demonstrated strong grasping abilities, remaining stationary even in strong winds. The oviposition process was rarely interrupted by light, sound, or slight touches. A few ovipositing females could glide down from trees when forced to stop laying eggs, but the flight distance was short, and it was difficult for them to take flight again (i.e., no upward displacement). After landing on branches or trunks, they continued ovipositing.

#### 3.4.3. Verification of Phototactic Flight Behavior in the Laboratory

After exhibiting a phototactic response, *L. xylina* adults displayed various modes of displacement, including flying, crawling, and wing flapping. Displacement could be a single mode or a combination of multiple modes. Displacement results were classified into two categories: (1) flying toward the lamp directly (i.e., direct displacement) and (2) not flying directly toward the lamp (i.e., moving near the lamp but deviating from it, not taking off, or not flying towards the lamp).

In Test 1, out of ten female moths, four exhibited a phototactic response. Among these, one female moth displaced toward the lamp directly (displacement involved flying and crawling while flapping its wings but failed to enter the lamp due to a flight height below 35 cm).

In Test 2, out of ten female moths, two exhibited a phototactic response, flapping their wings but without displacement. Out of ten male moths, five exhibited a phototactic response, with three flying directly to the lamp.

#### 3.4.4. Field Mark–Release–Recapture in the Field

The results of the field mark–release–recapture experiment is shown in Table 5. No female moths were recaptured, and the recapture rates of male moths released at 30, 60, and 90 m from the lamp were 6.7%, 3.3%, and 3.3%, respectively.

## 4. Discussion

Theoretically, *L. xylina* in Fuzhou presents the risk of long-distance dispersal via ocean-going cargo vessels due to its ability to fly towards light sources (phototaxis) and lay eggs on various surfaces, including artificial objects. The prevalence of *Casuarina* trees, a preferred host for the insect, in Fuzhou’s coastal shelter forests, combined with the proximity of ports to these forests, creates ideal conditions for dispersal to other regions through ocean-going freighters in coastal ports [24,25,26]. Limited evidence suggests that some non-ovipositing female *L. xylina* exhibit phototactic behavior and are capable of short-distance flights, with a maximum uninterrupted flight distance of up to 34.5 m and a total flight distance of up to 184.5 m in the field. This indicates the potential for females to fly towards the ports, especially if attracted by light sources. Additionally, their ability to lay eggs on artificial objects, such as the wooden door, suggests the possibility of oviposition on freighters or cargo at ports. Since 2015, a small number of male and female *L. xylina* moths have been reportedly intercepted on ships entering Korean ports [1,11,12,13], and egg masses have also been intercepted on vessels called at the US ports during 2014–2016 (Wang, B. unpublished data), indicating *L. xylina* adults may have the potential to fly to ocean-going freighters located at ports and disperse over long distances via ocean-going freighters. However, it remains uncertain whether these moths originated from mainland China. The flight behavior of non-ovipositing female *L. xylina* exhibits a pronounced circadian rhythm, with peak flight around 20:00. Once egg laying begins, they cease flight. Previous studies have shown that gravid female *L. xylina* prepare for flight during the evening (from sunset to around 21:00), and 91.2% of the 110 females were collected in the evening were not mated [10]. This suggests that the majority of active flying females are non-ovipositing, primarily unmated. For the related *L. dispar japonica*, the flight behaviors of females were observed in a net cage under natural photoperiodic conditions after they were marked on their forewings. Both unmated and mated (but not egg-laying) females fly between 19:00 and 21:00. If females fail to mate during the day, they will fly again in the evening to find a mating site. Those that have mated (but not laid eggs) during the day typically fly to egg-laying sites after sunset and do not move once they start laying eggs. If female moths mate at night, they usually begin laying eggs at the same site without flying [27]. Thus, it is inferred that the general status of active flying female *L. xylina* is non-ovipositing, primarily unmated, with the main flight time being 19:00~21:00. Yeh et al. used high-pressure mercury lamps to trap adult *L. xylina* in the *lychee* forest of Changhua County, Taiwan, observing that the adults began to fly at 19:19, with the number of females caught peaking at 19:30, and female moths rarely moved again after 20:10 [28]. Similarly, five types of fluorescent insecticidal lamps were used to trap *L. xylina* adults in the *Casuarina* plantation in Fuzhou from 4 June to 6 June 2019, and the trapped rhythm of the female *L. xylina* was obvious, with most females caught during 19:00–20:00 and none caught after 23:00 (unpublished data). Both examples indicate that females are more sensitive to light sources in the evening (19:00–20:00). The peak courtship period of the female moth was around 20:00, similar to the peak flight period (19:00–20:00) and the peak time flying toward the lamp directly (19:00–20:00). Therefore, it is concluded that non-ovipositing female *L. xylina* is attracted by the light of the port when flying in search of mating or egg-laying site and then fly to the freighter or cargo to wait for mating or oviposition. The main phototaxis flight time is 19:00–21:00.

Compared with *L. dispar*, female *L. xylina* do not prefer artificial objects as egg-laying substrates, and the viability of eggs laid on objects is generally lower. In the absence of *Casuarina* twigs as oviposition sites, the number of eggs laid by female moths decreased significantly. In the ship’s cabin simulation tests, *L. xylina* laid few and small egg masses with low egg counts. Under natural conditions, *L. xylina* do not prefer to lay eggs on artificial objects [29,30,31], but they prefer columnar objects among artificial objects. These observations align with the distribution of *L. xylina* egg masses in nature. After mating, females typically lay eggs on the host or nearby vegetation, primarily on twigs or branches [9,29]. The preferred egg-laying positions on *L. chinensis* were branches less than 2 cm in diameter [30], while on *Casuarina* trees, they were twigs and lateral branches (97.03%), with a diameter usually less than 2 cm and a height between 240 and 360 cm [31]. Females of herbivorous insects, such as *Leptinotarsa decemlineata* Say, use chemicals released by the host plant to find their hosts, resulting in host-oriented behaviors. Lepidopteran insects often mate and lay eggs in host-intensive places so that larvae can quickly find food upon hatching. Ovipositing females may delay or reduce mating behavior until they locate a suitable host, as observed in *Antheraea polyphemus* Cramer [32,33]. To expose egg masses to higher temperatures, *L. dispar* prefer to lay eggs on vertical surfaces, such as the trunk epidermis and lateral branches. They may also lay eggs on flat objects such as square stone pillars, pavilions, street lamp poles, and cement-imitation wooden guardrails. The egg masses are clustered together, and the number of eggs laid on these objects is similar to the number of eggs laid on the host [8,22]. Unlike *L. dispar*, *L. xylina* egg masses are less susceptible to temperature fluctuations, although they are not resistant to extreme high temperatures, with a mortality rate of 95.6% at 50 °C for 3 h, and 100% at 40 °C for 5 days. Similarly, the egg masses of *L. xylina* also struggled to survive in extremely low temperatures, with a mortality rate of 89.8% at −15 °C for 1 d and 98.5% at −10 °C for 5 d (unpublished data).

Egg masses laid by light-attracted females may be unfertilized or incompletely fertilized. The sex ratio of *L. xylina* is skewed against females (0.33), and there are frequent instances of secondary mating by females. To ensure sufficient males for mating, lepidopteran males and females generally engage in multiple mating, which can improve fertility and fecundity [34,35,36]. In the case of *L. dispar*, females that are not fully fertilized in the first mating may remate after two days and be fertilized using sperm from the second mating [37,38]. The average hatching rate of *L. xylina* egg masses is 84.4% (47.7–98.9%), while the highest parasitism rate is only 3.1% (mainly parasitism by *Ooencyrtus kuvanae* Howard) [21,39]. Some unhatched egg masses were observed in the field during light-trapping tests conducted in 2019 and 2020 in Fuzhou, Fujian Province. These observations suggest that non-fertilization or incomplete fertilization is a common phenomenon in *L. xylina*. Female *L. xylina* often need to mate twice or even multiple times to complete the fertilization of all eggs. It has been reported that 91.2% of female *L. xylina* flying in the evening were unmated [10]. This phenomenon is closely related to their behavioral strategy (circadian rhythm), with emergence mostly occurring during the day and mating mostly occurring around midnight. A similar pattern is observed in *Lymantria mathura* Moore, a close relative of *L. xylina*. Emergence occurs in the afternoon, copulation at nightfall, and most female *L. mathura* were unmated during phototactic flight in the evening [40]. This may help explain why most of the females collected in the evening are unmated. In contrast, the peak emergence period of female *L. dispar* asiatica is 10:00–11:00, the peak mating period is 14:00–16:00, and most female *L. dispar* asiatica had copulated (not laid eggs) before phototactic flight in the evening [23,40,41]. Additionally, light disturbance can deepen the degree of non-fertilization of *L. xylina* eggs because light interferes with the recognition and mating of male and female moths, reducing the chance of finding a mating mate. Moreover, when most male moths are attracted by light, the existence of female moths is ignored, as sex pheromones may not be attractive to male moths at this time [42,43,44].

The flight ability of female *L. xylina* is relatively weak compared to that of female *L. dispar asiatica* or *L. dispar japonica*. Limited data indicate that the maximum average flight speed of non-ovipositing *L. xylina* females in the field under stress was 12.7 m/min, with a maximum total flight duration of 14.5 min and a total flight distance of 184.5 m. In contrast, the flight speed of female *L. dispar japonica* in cages was estimated to be 21.3 m/min, with unmated females flying up to 35 min and mated females up to 25 min, with maximum flight distances of 746 m and 511 m, respectively [27,41,45]. Despite differences in research methods, and the extraordinary flying ability of non-ovipositing *L. xylina* females due to their escape mechanism [46,47], it is evident that the overall flight capabilities of non-ovipositing *L. xylina* females are weaker than those of *L. dispar* asiatica or *L. dispar japonica* females. However, tethered to a flight mill for 24 h, female *L. xylina* demonstrated a higher potential flight ability, with an average maximum uninterrupted flight distance of 651 m. One-day-old females exhibited the strongest potential flight ability, with average flight speed, total flight distance, total flight duration, distance per flight, duration per flight, and number of flights of 25.6 m/min, 3975 m, 111.1 min, 63 m, 15.6 min, and 86 times, respectively [48]. Compared to their potential flight ability, the flight ability in nature is weaker, and the flight duration in the cage is shorter. The descending speed of female *L. xylina* is mainly determined by its weight and the lift generated by flapping, which in turn determines the flight duration [49]. When tethered to a flight mill, the female needs to overcome less of its own weight to fly, even if it does not have enough lift to fully overcome gravity during wing flapping. As a result, the flight parameters during the tethered flight are greater than those in the actual flight, indicating that the potential flight ability exceeds the flight capability. In addition, the flight period of female *L. xylina* was relatively short, with a flight season of 35 days, a peak flight period of 19:00–21:00, and a lifespan of 4.5 days. Once females start laying eggs, they no longer fly actively. The flight of non-ovipositing female moths is dominated by short-term intermittent flight with a low flight altitude, characterized by a long preparation time, fast landing, and a short continuous flight distance. These flight characteristics based on our limited data suggest that *L. xylina* females may be less likely to undertake long-distance continuous flights. However, more extensive research with larger sample sizes is needed to confirm these findings, and there may be populational or individual variations or specific environmental conditions that allow for exceptions.

The fecundity of strong-flying females may be relatively low. In the limited field tracking observation, four female moths did not fly, and one female moth only glided. Visual observations revealed variations in body sizes among the remaining three females, and their total flight distances differed. Small female moths can fly quickly, but over short distances. Flight mill tests also demonstrated that female moths with the longest total flight distance were the lightest and laid the fewest eggs [48]. Extensive evidence suggests that flying and non-flying adults can co-exist in the same species, with a trade-off between flight ability and reproductive capacity [22,50,51,52]. Reports indicate that some of the female *L. dispar dispar* in North America, considered flightless, may perform short-distance flights sometimes. Among these flying females, body sizes tend to be smaller than those of non-flying females [53,54]. In Hokkaido, Japan, *L. dispar japonica* females are smaller than those in Honshu, Shikoku, and Kyushu, and their flight distance is the longest [41]. Additionally, in 2019, the weight and egg-carrying capacity of the field light-trapped group (0.75 ± 0.05 g, 383.8 ± 32.3 eggs) were significantly lower than those of the control group (1.03 ± 0.06 g, 685.2 ± 42.4 eggs) in the laboratory (unpublished data). The weight of the male pupae of *L. dispar dispar* ranged from 0.19 to 0.73 g, with an average of 0.41 ± 0.013 g [55,56]. Like *L. dispar*, the weight of male and female pupae of *L. xylina* also varies greatly (0.74 ± 0.14 g for males and 1.73 ± 0.35 g for females) [57]. Similarly, the weight of male and female moths of *L. xylina* is quite different, especially among the females, which exhibit a large difference in the number of eggs laid (12–1330 eggs). Based on the biological significance, a strong linear relationship exists between the body weight and fecundity of females; that is, their fecundity increases with an increase in body weight, and their body weight is closely related to their body size. The body size of insects is the biggest factor affecting flight ability, which is usually related to the indicators of flight ability [55,58,59,60]. Therefore, it is generally believed that *L. xylina* females reaching freighters via phototactic flight may be lighter, smaller, and less fecund.

In summary, *L. xylina* in Fuzhou theoretically poses a risk of long-distance dispersal via ocean-going freighters through phototactic flight and oviposition. However, the risk is relatively lower than *L. dispar asiatica* and *L. dispar japonica* based on its reproductive and flight characteristics. While the risk from the Fuzhou population may be relatively low, the potential risk from other geographic populations cannot be excluded. *L. xylina* exhibits significant geographic variation in its adult weight, forewing length, and sex ratio due to differences in host plants and environmental conditions [29,30]. Additionally, the morphology of different *L. xylina* subspecies varies, with gradual changes in body size and flight ability from west to east [61,62]. Within *L. xylina* subspecies, there are also significant differences in flight ability, flight tendency, and morphological characteristics (weight, wing length, wing area, aspect ratio, load ratio, etc.) among different geographic populations [63,64,65]. These variations suggest that the dispersal potential of *L. xylina* may differ among geographic populations. Further research is needed to assess the risk posed by *L. xylina* from various regions.

## 5. Conclusions

This study investigated the dispersal risk of *L. xylina* adults in Fuzhou based on their reproductive and flight characteristics. We found that while *L. xylina* adults in Fuzhou, China, theoretically possesses the potential for long distance dispersal via ocean-going freighters, several factors may limit its ability to establish new populations.

Flight Ability: *L. xylina* females exhibit relatively weak flight abilities, making long-distance dispersal challenging.

Oviposition Preferences: *L. xylina* generally prefers to lay eggs on host plants, and eggs laid on artificial objects may have low fecundity.

Reproductive Limitations: Unfertilized or incompletely fertilized eggs, coupled with the need for multiple mating, can reduce reproductive success.

While the risk from the Fuzhou population may be relatively low, the potential for long-distance dispersal by *L. xylina* from other geographic populations cannot be excluded.

Further research is needed to assess the dispersal risk of *L. xylina* from various geographic regions and develop effective risk mitigation and control strategies.

## Figures and Tables

**Table 1:** The effects of different times and dates on the emergence of female and male *L. xylina* in 2020.

Source	Female			Male		
	df	F	*p*	df	F	*p*
Date	7	1225.16	<0.001	8	3415.928	<0.001
Time	13	21.405	<0.001	19	68.899	<0.001
Date × time	18	1.465	0.196	26	10.43	<0.001
Error	22			69		
Total	61			123		

**Table 2:** Weight statistics of male and female *L. xylina* in the laboratory in 2020.

Sex	Number	Min.	Max.	Mean	SD	SE	Kurtosis	Skewness
Male	123	0.07	0.30	0.16	0.05	0.004	0.40	−0.06
Female	61	0.27	1.30	0.74	0.24	0.03	0.21	−0.38

**Table 3:** Effect of oviposition location on the egg laying of *L. xylina* adults in the laboratory in 2021.

Oviposition Location	Number of Ovipositing Females			Pre-Oviposition Period (h)	Oviposition Duration (h)		Number of Eggs
	≥200	1–200	0				
*Casuarina* twigs	3	3	3	31.4 ± 8.4 a	21.0 ± 6.3 a	429 ± 165 a	
No twigs	1	2	6	32.0 ± 7.6 a	4.3 ± 1.4 b	164 ± 77 b	

Note: The data in the table are expressed as the mean ± SE, and different lowercase letters represent a significant difference between the two groups (*p* < 0.05, *t*-test).

**Table 4:** Field observation of flight ability of non-ovipositing *L. xylina* females in Fuzhou in 2022.

Individual No.	Total Flight Distance (m)	Total Flight Duration (min)	Flight Speed (m/min)	Number of Flights	Distance per Flight (m)	Duration per Flight (min)
1	71.0	6.9	10.3	9.0	7.9	0.8
2	184.5	14.5	12.7	21.0	8.8	0.7
3 *	1.5	0.1	15.0	1.0	1.5	0.1
4	17.0	2.6	6.5	12.0	1.4	0.2

* The moth glided down from the tree and could not take off.

**Table 5:** The results of mark–release–recapture testing of *L. xylina* adults from 15 June to 17 June 2021, in Fuzhou, China.

Date	Mark Color	Direction	Mark Position	Distance (m)	Number of Releases	Number of Recaptures
15-Jun	Red	East	Lateral forewing	90	10 males, 5 females	0
	Black	West			10 males, 5 females	1 male
	Blue	South			10 males, 5 females	0
16-Jun	Red	East	Hindwing	60	10 males, 5 females	1 male
	Black	West			10 males, 5 females	0
	Blue	South			10 males, 5 females	0
17-Jun	Red	East	Medial forewing	30	10 males, 5 females	1 male
	Black	West			10 males, 5 females	1 male
	Blue	South			10 males, 5 females	0

## Data Availability

Data are contained within the article.

## Supplementary Materials

The following supporting information can be downloaded at https://www.mdpi.com/article/10.3390/insects15110894/s1: Figure S1: Milk carton trap; Figure S2: Ship’s cabin simulation test; Figure S3: The main investigation of attachment of *L. xylina* egg masses on objects in 2017; Figure S4: The female *L. xylina* was forced to fly by continuously knocking on the branch or trunk; Figure S5: The main copulation postures of *L. xylina*; Figure S6: Two male *L. xylina* aggregating around one female *L. xylina*.
