# Supplementary material for: Reproductive and Flight Characteristics of Lymantria xylina (Lepidoptera: Erebidae) in Fuzhou, China
Source: Insects. 2024 Nov 15;15(11):894. doi: 10.3390/insects15110894 (PMC11594333; doi:10.3390/insects15110894)
Supplement: Supplementary file 1 [file insects-15-00894-s001.zip › insects-3281488-supplementary.pdf]

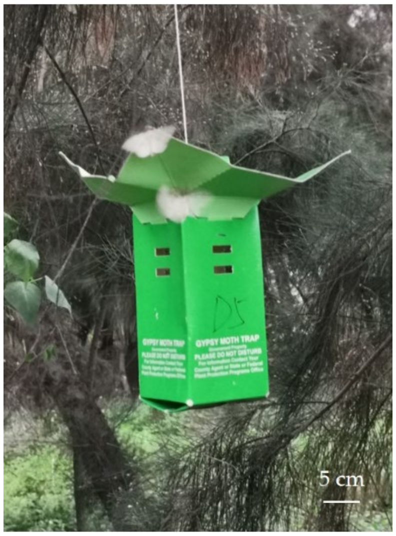

Figure S1. Milk carton trap.

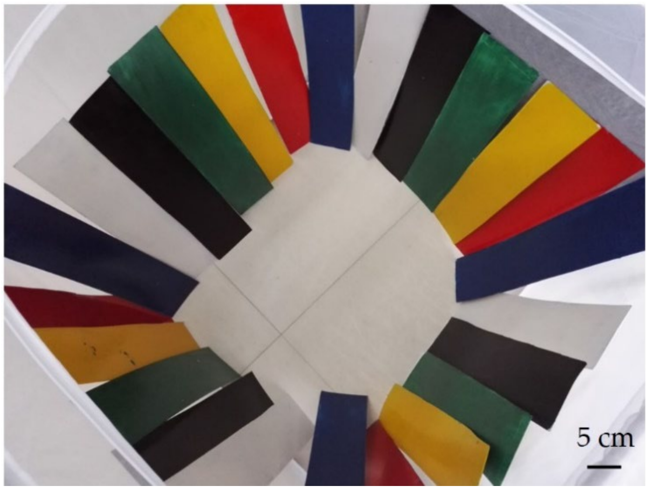

Figure S2. Ship’s cabin simulation test.

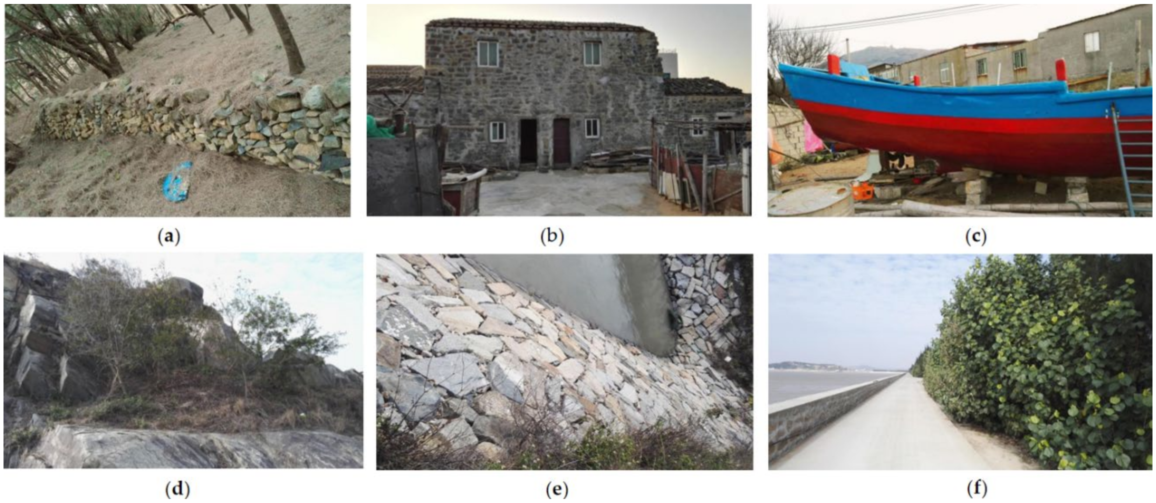

Figure S3. The main investigation of attachment of *L. xylina* egg masses on objects in 2017: (**a**) stones, (**b**) house, (**c**) boat, (**d**) hillside, (**e**) dam, (**f**) seawall.

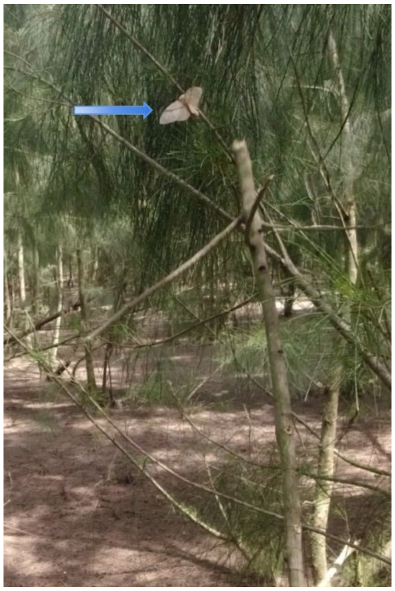

Figure S4. The female *L. xylina* was forced to fly by continuously knocking on the branch or trunk.

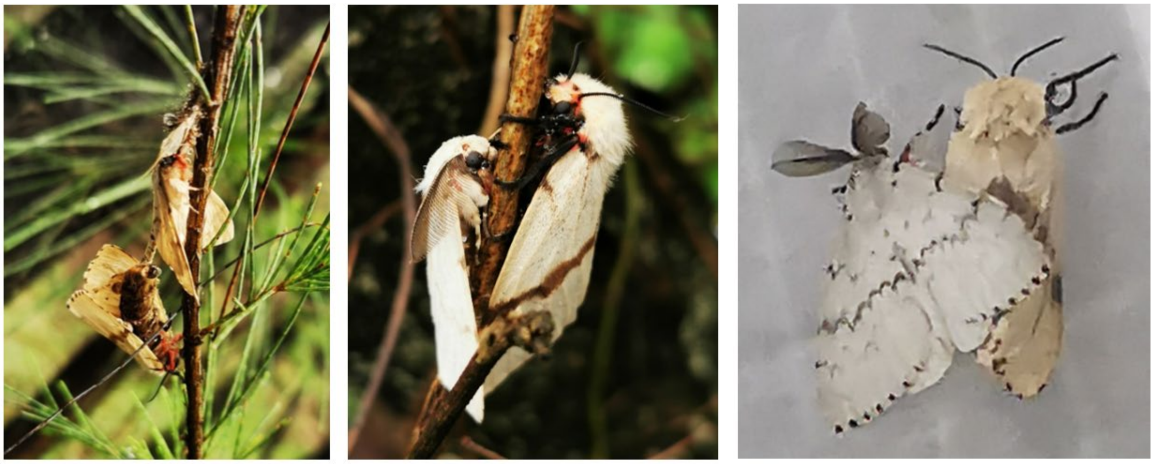

(**a**) (**b**) (**c**)

Figure S5. The main copulation postures of *L. xylina*: (**a**) end-to-end pose, (**b**) venter-to-venter pose, and (**c**) male on top with female dorsum up pose.

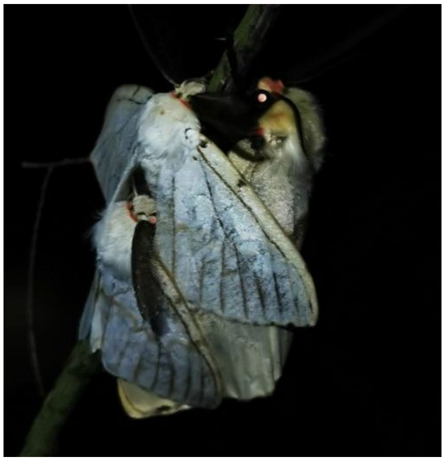

Figure S6. Two male *L. xylina* aggregating around one female *L. xylina*.
